# Additional Therapy with a Mistletoe Product during Adjuvant Chemotherapy of Breast Cancer Patients Improves Quality of Life: An Open Randomized Clinical Pilot Trial

**DOI:** 10.1155/2014/430518

**Published:** 2014-02-20

**Authors:** Wilfried Tröger, Zdravko Ždrale, Nevena Tišma, Miodrag Matijašević

**Affiliations:** ^1^Clinical Research Dr. Tröger, Zechenweg 6, 79111 Freiburg, Germany; ^2^Institute of Oncology and Radiology of Serbia, Pasterova 14, 11000 Belgrade, Serbia

## Abstract

*Background*. Breast cancer patients receiving adjuvant chemotherapy often experience a loss of quality of life. Moreover chemotherapy may induce neutropenia. Patients report a better quality of life when additionally treated with mistletoe products during chemotherapy. *Methods*. In this prospective randomized open-label pilot study 95 patients were randomized into three groups. All patients were treated with an adjuvant chemotherapy. The primary objective of the study was quality of life, the secondary objective was neutropenia. Here we report the comparison of HxA (*n* = 34) versus untreated control (*n* = 31). *Results*. In the explorative analysis ten of 15 scores of the EORTC QLQ-C30 showed a better quality of life in the HxA group compared to the control group (*P* < 0.001 to *P* = 0.038 in Dunnett-T3 test). The difference was clinically relevant (difference of at least 5 points, range 5.4–12.2) in eight of the ten scores. Neutropenia occurred in 7/34 HxA patients and in 8/31 control patients (*P* = 0.628). *Conclusions*. This pilot study showed an improvement of quality of life by treating breast cancer patients with HxA additionally to CAF. Although the open design may be a limitation, the findings show the feasibility of a confirmatory study using the methods described here.

## 1. Introduction

Quality of life of cancer patients is frequently reduced during and after chemotherapy [1]. But physicians have reported better quality of life in breast cancer patients additionally treated with mistletoe products during chemotherapy, compared to patients receiving chemotherapy alone or together with a placebo [2–5]. A systematic review of clinical trials in breast cancer patients [6] identified nine studies in which mistletoe products were given additionally to conventional chemotherapy, thereof three retrolective studies [7–9] and six randomized clinical trials (three are open-label [2, 3, 10] and three double-blind [4, 5, 11]). In one study, data on natural killer-cell activity and quality of life assessed by EORTC QLQ-C30 was collected. In all other studies disease- or therapy-related symptoms or quality of life, assessed by different questionnaires, were documented and showed an improvement favouring the additional therapy with mistletoe.

Because 70% of cancer patients use mistletoe products in Germany, randomized clinical trials are difficult to conduct: low recruitment rates and noncompliance because of therapy preferences are the consequences [11–14]. Therefore, we conducted this trial in Serbia, a country where mistletoe therapy was unknown.

This randomized clinical pilot trial was carried out to assess the effects of two different mistletoe products on quality of life and on neutrophil count when administered during CAF chemotherapy for breast cancer. The aim was to identify promising outcome measures and methods for future studies in Serbia and to use the results for sample size calculation for a following confirmative study. Here we present the results with the mistletoe product Helixor A (HxA).

## 2. Methods

### 2.1. Objectives

The objectives of this pilot study were to determine the clinical response (primary: quality of life, and secondary: neutropenia) of breast cancer patients to an additional mistletoe therapy during CAF. Our primary hypotheses were: breast cancer patients receiving mistletoe products during six cycles of consecutive treatment with CAF will show a better quality of life and less neutropenia compared to patients receiving CAF alone.

### 2.2. Design

We conducted a prospective randomized open-label pilot study with equal-size randomization into three groups. All three groups received six cycles of CAF. In addition, one group received Helixor A (HxA), another group received Iscador M, and a control group had no additional therapy. The study was not placebo-controlled because currently no active placebo is known that can imitate the typical and time-dependent reactions following subcutaneous injections of mistletoe products (reactions at the injection site, increased body temperature, and flu-like symptoms). Here we report the comparison of HxA versus control. The other part of this combined pilot study has been published elsewhere [10].

### 2.3. Participants

At the Institute of Oncology and Radiology, National Cancer Research Centre of Serbia in Belgrade (IORS), breast cancer patients in the stages T_1–3_N_0–2_M_0_ scheduled to receive six consecutive cycles of CAF after surgery were assessed for eligibility.

Additional inclusion criteria were female gender, age ≥ 18 years, Karnofsky-Index ≥ 60, leukocytes ≥ 3,000/mm³, thrombocytes ≥ 100,000/mm³, serum creatinine ≤ 2 mg%, serum glutamic oxaloacetic transaminase (SGOT), and serum glutamic pyruvic transaminase (SGPT) ≤ 2.5 × the upper institutional limits.

Exclusion criteria were pregnancy or lactation, distant metastases, planned radiation or hormone therapy during the CAF treatment period, use of immunostimulant or immunosuppressive agents (e.g., corticosteroids) except for nausea and emesis, current use of other investigational agents, clinically relevant physical or mental illness such as serious infections, hepatic, renal or other organ dysfunction, or major depression, alcohol abuse, alcoholism, oral or parenteral drug abuse, and methadone treatment.

### 2.4. Randomization

The chance to be allocated to any of the three groups (HxA, other mistletoe product, and control) was 1 : 1 : 1, for randomization variable block sizes were used. No stratification took place prior to randomization. The randomization sequence was generated by Clinical Research Dr. Tröger (CRDT), using SPSS (SPSS 14.0.1, SPSS Inc., Chicago, Ill, USA). Allocation concealment was implemented by using sealed envelopes, prepared by CRDT. Patients were enrolled by investigators at the Outpatient Clinic of the IORS, while the sealed randomization envelopes were stored in the Department of Study Coordination of the IORS and released consecutively for each enrolled patient.

### 2.5. Interventions

CAF was administered in six cycles with a three-week interval between each cycle. The scheduled dosage was 500 mg cyclophosphamide, 50 mg adriamycin, and 500 mg 5 FU per 1 m² skin surface administered at one day. All patients received antiemetic therapy with a single dose of ondansetron chloride 8 mg, dexamethasone 8 mg, and ranitidine 50 mg, respectively, administered prior to each CAF cycle.

No other antineoplastic or immunomodulatory therapies were permitted during the study. Patient compliance was examined by account of the questionnaires and patient diaries and at each visit.

Patients randomly allocated to the first group with additional therapy received Helixor A (HxA; aqueous extract of *Viscum album* from fir tree (*Abies alba*), fresh plant material). HxA was manufactured and provided by Helixor Heilmittel GmbH & Co. KG, Rosenfeld, Germany, and prepared in 1 mL ampoules for injection, each ampoule containing aqueous extract of 1, 5, 10, 20, 30, or 50 mg of fresh mistletoe herb, respectively, in isotonic saline solution. HxA was administered by subcutaneous injection of 1 mL HxA into the upper abdominal region three times per week. The patients were instructed to inject HxA themselves. The dosage of HxA followed the guidelines of the manufacturer with a stepwise increase: 3 × 1 mg, 3 × 5 mg, 3 × 10 mg, 3 × 20 mg, 3 × 30 mg, and remaining doses 50 mg. Dose-dependent inflammatory reactions at the injection site (redness and swelling, sometimes accompanied by itching) were monitored. If such reactions exceeded 5 cm in diameter, the dosage was decreased or the therapy was paused until the reactions had ceased. Depending on the beginning of the injections in the first week, 52–54 injections of HxA were planned. An average of 52.3 ± 2.8 injections with altogether 2,813 mg (min = 1 mg; max = 6,818 mg of HxA per patient) were administered in the HxA group.

### 2.6. Outcomes

The primary objective of the study was quality of life and the secondary objective neutropenia. Quality of life was documented with the European Organization for Research and Treatment of Cancer Quality of Life Questionnaire (EORTC QLQ-C30) in the official Serbian translation [15]. The EORTC QLQ-C30 has 30 questions and is analysed in 15 scores: six scores for functioning and nine symptom scores. Before each of the six CAF cycles and at least three weeks after the last CAF cycle, EORTC QLQ-C30 was filled in by the patients and the absolute number of neutrophils in the peripheral blood was determined. Neutropenia was defined as neutrophil count <1,000/*μ*L.

### 2.7. Assessment of Adverse Events

Adverse events (AE) were assessed by interviewing the patients and by analysing laboratory data at each visit. The Common Terminology Criteria of Adverse Events (v3.0) were used for grading. Local reactions to HxA less than 5 cm in diameter were expected reactions and therefore not classified as adverse events.

Neutropenia was one of the study objectives and was not classified as AE.

### 2.8. Statistical Methods

Statistical analysis (SPSS 14.0) was performed on the intention-to-treat population, a population consisting of all patients randomised into one of the three groups. Missing data were not replaced. Because of the pilot character of this study, all results of the analysis are explorative and do not have a confirmatory character. Therefore no sample size calculation was performed. A sample size of 90 patients (30 per group) was considered to be sufficient. For calculation of the EORTC QLQ-C30 scores, missing data within one scale were replaced according to the manual [16].

Quality of life (EORTC QLQ-C30) was analysed as follows: for each EORTC QLQ-C30, the mean change from baseline during follow-up in each group was compared among the HxA group, the group with the other mistletoe product, and the control group, using nonparametric marginal models according to Brunner and Langer [17] with therapy as whole-plot factor and time as subplot factor and a possible interaction between these two factors (results not shown). As a sensitivity analysis, a parametric covariance pattern model was also applied and found to qualitatively concur with the nonparametric results. For a more comprehensible presentation, the estimates of this parametric model will be shown: post-hoc analyses of differences between the HxA group and the control group were performed using the Dunnett-T3 test. Clinical relevance of between-group differences of EORTC QLQ-C30 scores was classified according to Osoba et al. [18] as small (5–10 points), moderate (11–20 points), and large (>20 points).

For the incidence of neutropenia, the difference between the HxA and control groups was analysed by chi-square test according to the sequential rejective Holm procedure.

### 2.9. Adherence to Regulations and Guidelines

The study was approved by the Ethics Committee of the National Cancer Research Centre of Serbia without modifications (date: 3 October 2005) and by the Serbian Drug Agency (date: 01 November 2005). Due to its pilot character, this study was not registered in a public study registry. The study was conducted in compliance with the Declaration of Helsinki, Good Clinical Practice guidelines, and national laws. A patient insurance was provided for all participants. All patients provided signed informed consent prior to inclusion. CRDT was responsible for planning, conduct, monitoring, and analysis of the study. Two audits at the CRDT office and one at the study site were performed by the two sponsors during the study; no violation of Good Clinical Practice was detected.

This publication followed the CONSORT-PRO statement for reporting of randomized trials [19], therefore “standardized mean differences” (effect sizes) are calculated [20].

## 3. Results 

### 3.1. Recruitment, Participant Flow, Assessment, and Numbers Analysed

**Figure 1:**
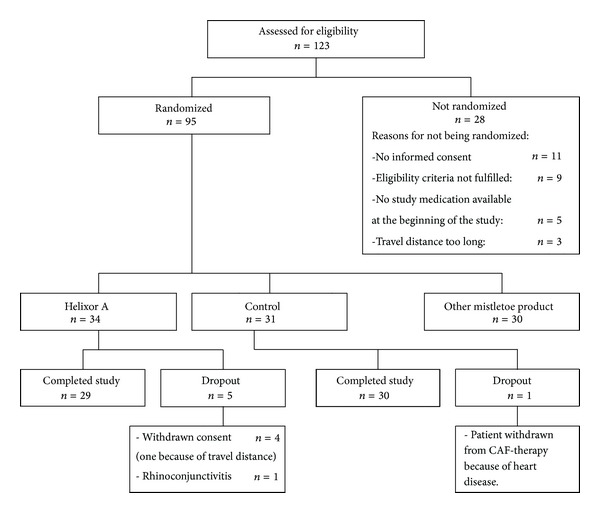
Detailed flow chart of the patient disposition.

From 14 December 2005 to 15 February 2007 a total of 123 breast cancer patients were scheduled for treatment with CAF and assessed for eligibility at the IORS study centre. 28 patients did not fulfil the eligibility criteria (reasons: see Figure 1), whereas 95 patients were included and randomized into the 3 therapy groups: CAF and HxA (*n* = 34), CAF and another mistletoe product (*n* = 30), and CAF without additional therapy (*n* = 31). One patient in the control group was withdrawn from further CAF therapy after three cycles of CAF because of heart disease (Figure 1).

In the HxA group, 5 patients dropped out; reasons were withdrawal of informed consent (*n* = 4, in one of these patients travel distance to study centre was the reason) and rhinoconjunctivitis with putative relationship to mistletoe therapy (*n* = 1). These dropout patients were replaced to achieve at least 30 in both groups. All other patients in the HxA group and all patients in the control group received the six scheduled CAF cycles.

The EORTC QLQ-C30 was evaluable for 86.9% (207 of 238) of planned visits in the HxA group, and for 97.2% (211 of 217) of planned visits in the control group. None of the expected questionnaires were missing in the follow-up phase of the study (Table 1). The neutrophil count was determined at 88.6 % (211 of 238) and 98.2 % (213 of 217) of planned visits in the HxA group and control group, respectively.

### 3.2. Baseline Data of the Patient Groups

The HxA group and the control group did not differ significantly regarding age, tumour stage, body mass index, physical status, vital signs, previous diseases, EORTC QLQ-C30 scores, and neutrophil counts (Table 2).

### 3.3. Quality of Life during Chemotherapy

During chemotherapy with CAF, a deterioration of the quality of life occurred in 14 of 15 EORTC QLQ-C30 mean scores in the control group of which 4 were clinically relevant; whereas a deterioration occurred in 5 of 15 scores in the HxA group, of which 2 were clinically relevant.

**Figure 2:**
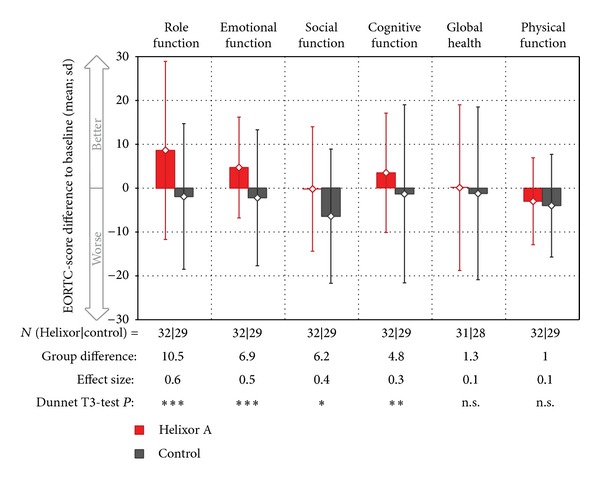
Differences of the mean of six follow-up values to baseline values of the EORTC QLQ-C30 function scores (mean ± sd), sorted by between-group group differences. All patients with at least one follow-up visit are displayed. *P* values are corrected using Bonferroni's method regarding 15 EORTC scores tested and defined as follows: ***P < 0.001 (extremely significant); **P < 0.01 (highly significant); *P < 0.05 (significant); n.s.: not significant.

**Figure 3:**
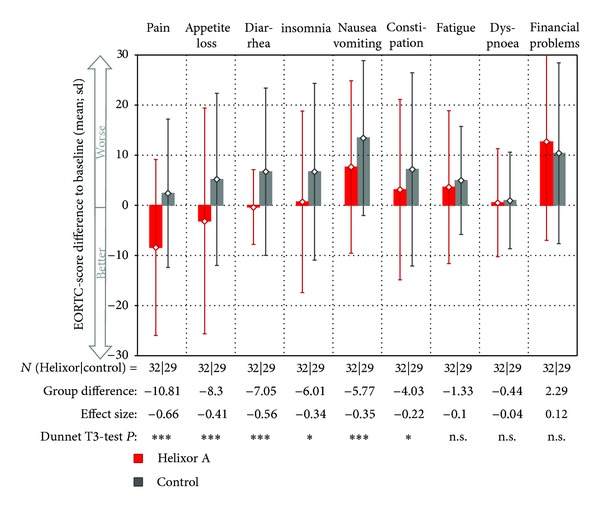
Differences of the mean of six follow-up values to baseline of the EORTC QLQ-C30 symptom scores (mean ± sd) sorted by group differences. All patients with at least one follow-up visit are displayed. *P* values are corrected using Bonferroni's method regarding 15 EORTC scores tested and defined as follows: ***P < 0.001 (extremely significant); **P < 0.01 (highly significant); *P < 0.05 (significant); n.s.: not significant.

In the adjusted analyses, mean differences from baseline were compared between the two groups for each EORTC QLQ-C30 score: 14 of 15 comparisons favoured the HxA group and one comparison (financial difficulties) favoured the control group. Ten of 14 comparisons favouring the HxA group showed significant differences (Figures 2 and 3). Eight of these 10 significant between-group differences of the EORTC QLQ-C30 scores were clinically relevant (>5 points), of which two (Pain −10.8 points, Role function +10.5 points) were moderate and the remaining six were small (5–10, Figures 2 and 3).

In explorative analyses, the differences from baseline for each of the 15 scores at each of the six follow-up assessments were compared between the two groups. 80 out of 90 differences favoured the HxA group, while 10 differences favoured the control group (data not shown). Furthermore, moderate differences of at least 10 points were found once in nine scores (role function, emotional function, social function, and global health: pain, insomnia, nausea/emesis, appetite loss, and diarrhoea), small differences of 5–10 points were found in three scores (cognitive function, fatigue, and constipation), and differences <5 points were found in the remaining three scores.

### 3.4. Neutropenia during Chemotherapy

Neutropenia (neutrophil count < 1,000/*μ*L) was detected seven times in seven different patients of the HxA group and nine times in eight different patients of the control group (Table 3) (*P* = 0.628).

### 3.5. Adverse Events

Altogether 1*‚*527 injections of HxA were administered. Twenty of 34 patients (59%) of the HxA group reported altogether 45 adverse events (AE). The 45 AE were: local inflammatory reaction at the injection site > 5 cm, with definite causal relation to HxA (*n* = 42, representing 2.7% of 1,527 injections); rhinoconjunctivitis at one eye, documented by the investigator to be probably caused by HxA—the patient withdrew informed consent and gave no further information (*n* = 1); and AEs without any causal relationship to the administration of HxA (*n* = 2).

In the control group, 12 AEs and one serious adverse event (SAE: acute hospitalization because of dehydration upon severe emesis; see Table 4) occurred.

## 4. Discussion

In this randomized pilot study quality of life (EORTC QLQ-C30) and incidence of neutropenia were investigated in breast cancer patients undergoing adjuvant CAF chemotherapy. Patients receiving HxA in addition to CAF had significantly better quality of life, compared to patients receiving CAF alone. HxA therapy was well tolerated.

Strengths of this study include a high recruitment rate, detailed assessments of therapy implementation, high therapy compliance, and very low dropout rates.

Due to the open-label design, the study cannot distinguish between direct drug effects on quality of life and possible indirect effects from therapy expectations, therapy administration, and so forth, in the HxA group.

Generally, medication trials are blinded to separate pharmacological effects from placebo effects. However, the lack of blinding may not necessarily have had relevant effects on the results of this study: an updated Cochrane review of randomized trials comparing placebo to no treatment found no significant placebo effects on eight out of ten evaluable indications, small effects on self-reported pain, and moderate effects on phobia. Even these effects might have been confounded by biases [21–23].

This study was designed as a pilot study, and the limited sample size of 30 patients per group does not allow for hypothesis confirmation. Moreover dropout patients or patients with missing data were more frequent in the HxA-group. As the results were calculated using data from all included patients (ITT), data of two patients in each group were missing. The remaining differences are additional three patients (ITT) in the HxA-group, which may lead to a biased result. Nevertheless, significant differences in 10 of 15 EORTC QLQ-C30 scores favouring the HxA group were found. All of these scores showed a clinically relevant difference of at least 5 points. The latter scores include role function, emotional function, pain, nausea, emesis, appetite loss, and diarrhoea, which are relevant symptoms in patients during chemotherapy with CAF.

These results are consistent with findings from another randomized phase III trial, wherein the administration of HxA to patients undergoing chemotherapy for breast cancer, lung cancer, or ovarian cancer was associated with significant beneficial effects on quality of life [3]. Notably, in the latter study, different methods for assessment of quality of life were used (“Functional Living Index-Cancer” questionnaire filled in by the patients, and Karnofsky performance index classified by the attending physician).

On the other hand, in a trial of early breast cancer patients receiving radiochemotherapy (CMF schedule), no influence of adjunctive therapy with HxA on any of the EORTC QLQ-C30 scores was found [11]. This discrepancy to the present study may be attributed to the fact that in the latter trial, patients did not experience any significant deterioration of the EORTC QLQ-C30 scores during radiochemotherapy. Consequently, a favourable effect of the additive mistletoe therapy could not be achieved.

On the whole, there is some evidence that mistletoe extracts may have an impact on quality of life and reduction of side effects of chemotherapy especially in case of breast cancer patients as concluded in two recent comprehensive systematic reviews [24, 25].

In this pilot study, the effects of HxA on quality of life increased over time with the largest effects observed at the last follow-up visit. A longer follow-up period might show additional long- term benefits of HxA therapy.

## 5. Conclusions 

In this randomized pilot study of patients receiving adjuvant CAF chemotherapy for breast cancer, additional treatment with mistletoe therapy (Helixor A) was associated with significant and clinically relevant improvements of quality of life. Although the open design may be a limitation, the findings show the feasibility and justification of a phase III study using the methods described here.

## Figures and Tables

**Table 1:** Baseline status.

	Group		*P* values
	HxA (*n* = 34)	Control (*n* = 31)	
Age in years (mean ± SD; *t*-test)	50.4 ± 6.9	50.8 ± 8.0	0.228
Tumor status (*n*; Chi square test)			
Tumor classification			0.619
T1	9	9	
T2	24	19	
T3	1	2	
T*x*	0	1	
Pos. lymph nodes			0.849
N0	16	16	
N1	16	14	
N2	2	1	
Tumor grade			0.474
G1	3	1	
G2	28	24	
G3	3	6	
Menopause status (*n*; Kruskal-Wallis test)			0.475
Premenopausal	18	13	
Perimenopausal	2	1	
Postmenopausal	13	17	
Unknown	1	0	
Receptor status estrogen			0.507
Positive	21	17	
Negative	9	12	
unknown	4	2	
Receptor status progesterone			0.620
Positive	22	19	
Negative	8	10	
Unknown	4	2	
Body Mass Index (mean ± SD; *t*-test)	26.1 ± 4.3	25.6 ± 4.7	0.709
Karnofsky-Index (mean ± SD)	100 ± 0.0	100 ± 0.0	—
Abnormal findings on physical examination (*n*)	0	0	—
Vital signs (mean ± SD; *t*-test)			
Blood pressure systolic (mmHg)	126.1 ± 12.9	132.2 ± 19.0	0.139
Blood pressure diastolic (mmHg)	79.9 ± 9.2	83.6 ± 13.6	0.209
Pulse (/min)	77.3 ± 13.0	77.1 ± 10.1	0.945
Temperature (°C)	36.6 ± 0.1	36.6 ± 0.1	1.000
Primary outcomes			
EORTC QLQ-C30 (mean ± SD; Mann-Whitney *U*-test)			
Global health status	66.9 ± 21.5	68.5 ± 18.3	0.713
Physical functioning	85.6 ± 14.6	86.0 ± 12.4	0.872
Role functioning	68.6 ± 23.5	73.0 ± 16.9	0.443
Emotional functioning	69.4 ± 17.9	74.1 ± 18.1	0.274
Cognitive functioning	82.8 ± 17.2	79.3 ± 23.4	0.23
Social functioning	73.5 ± 22.5	80.5 ± 18.9	0.226
Fatigue	25.5 ± 18.3	25.7 ± 19.5	0.894
Nausea and vomiting	7.4 ± 16.0	2.9 ± 7.8	0.285
Pain	21.1 ± 20.2	16.1 ± 20.6	0.273
Dyspnea	2.9 ± 9.6	3.4 ± 10.3	0.839
Insomnia	20.6 ± 26.0	23.0 ± 31.0	0.902
Appetite loss	14.7 ± 27.5	10.3 ± 20.1	0.704
Constipation	8.8 ± 18.9	6.9 ± 13.7	0.914
Diarrhoea	4.9 ± 14.5	2.3 ± 8.6	0.498
Financial difficulties	20.6 ± 23.2	20.7 ± 30.1	0.661
Secondary outcomes			
Incidence of neutropenia	0	0	—

**Table 2:** Number of patients and compliance in the completion of QoL forms by visit and groups.

Group Visit	Time schedule (days)	Time window (days)	Dropout	Forms expected	Forms received
Control					
1	0	0-0	0	31	29 (94%)
2	21	21–25	0	31	31 (100%)
3	42	42–49	0	31	31 (100%)
4	63	63–74	1	30	30 (100%)
5	84	84–98	1	30	30 (100%)
6	105	105–122	1	30	30 (100%)
7	182 ≥ *x* ≥ 26	126–143	1	30	30 (100%)

Helixor:					
1	0	0-0	0	34	34 (100%)
2	21	18–28	2	32	32 (100%)
3	42	39–51	5	29	29 (100%)
4	63	63–75	5	29	29 (100%)
5	84	84–96	5	29	29 (100%)
6	105	105–117	5	29	29 (100%)
7	182 ≥ *x* ≥ 126	126–155	5	29	29 (100%)

**Table 3:** List of patients experiencing a neutropenia.

Group	ID	Visit number	Date (Visit)	Age	Stage	T	N	M	G	Leuco-cytes/nL	Neutro-phils/nL
HxA	18	2	26.04.2006	43	2	2	1	0	2	10.2	0.6
	61	2	18.09.2006	50	2	2	0	0	2	2.6	0.9
	42	7	03.11.2006	47	2	2	1	0	2	1.4	0.5
	53	7	06.12.2006	44	2	2	1	0	2	1.6	0.4
	57	7	22.12.2006	64	2	2	1	0	3	1.5	0.3
	58	7	22.12.2006	37	2	2	0	0	3	2.5	0.7
	75	7	07.03.2007	55	2	2	0	0	2	3.2	0.9

Control	13	3	26.04.2006	32	2	2	1	0	2	2.8	0.9
	33	5	29.08.2006	60	2	2	0	0	2	2.4	0.9
	87	6	26.03.2007	45	2	2	1	0	2	3.3	0.3
	90	6	29.03.2007	52	2	1	0	0	3	2.6	0.8
	51	7	08.12.2006	66	2	2	1	0	2	1.3	0.3
	56	7	21.12.2006	53	2	2	1	0	2	3.5	0.9
	62	7	31.01.2007	62	2	1	1	0	2	2.5	0.8
	66	7	23.01.2007	44	1	1	0	0	2	2.4	0.8
	90	7	19.04.2007	52	2	1	0	0	3	2.7	0.9

Seven patients of the HxA group and eight patients of the control group experienced a neutropenia (*P* = 0.628; 2-sided Chi square test) at day 21 of the respective cycle/visit.

**Table 4:** List adverse events.

Group AE	Code*	Number	Grade**number		Relation	Duration	Outcome
Helixor A			Adverse events				
Localized skin reaction at the injection site	10022096	42	Mild Moderate Severe	1 13 27	Definite	Median 4 days (lq 2–uq 6,5)	Resolved without sequelae
Conjunctivitis	10010741	1	Moderate	1	Probable	Unknown	Unknown
Febrile temperature	10021113	1	Severe	1	Unrelated	2 days	Resolved without sequelae
Sting	—	1	Severe	1	Unrelated	Unknown	Unknown

Control			Adverse events				
Urticaria (localized)	10046749	1	Mild	1	Unrelated	3 days	Resolved without sequelae
Wound infection	10048038	1	Severe	1	Unrelated	6 days	Resolved without sequelae
Nausea/emesis	10028813	3	Severe	3	Unrelated	2 days	Resolved without sequelae

Control			Serious adverse events:				
Nausea/emesis***	10028813	1	Severe	1	Unrelated	4 days	Resolved without sequelae

*Code according to the Medical Dictionary for Regulatory Activities.

**Grading according to the Common Terminology Criteria of Adverse Events (v3.0).

***Patient was hospitalized to treat dehydration. According to the ICH 2A Guidelines hospitalization is defined as “serious adverse event”.

Abbreviations: lq: lower quartile; uq: upper quartile; ICH: International Conference on Harmonisation of technical requirements for registration of pharmaceuticals for human use.

## Abbreviations

5-FU: 5-Fluorouracil
AE: Adverse event
CAF: Cyclophosphamide, adriamycine, and 5-fluoro-uracil
CRDT: Clinical Research of Dr. Tröger
EORTC QLQ-C30: European Organization for Research and Treatment of Cancer Quality of Life Questionnaire
HxA: Helixor A
IORS: Institute of Oncology and Radiology of Serbia
ITT: Intention to treat
SAE: Serious adverse event.
